# The influence of temperature on the electrical resistivity of the cellular polypropylene and the effect of activation energy

**DOI:** 10.1186/2193-1801-2-472

**Published:** 2013-09-18

**Authors:** Floran Vila, Pranvera Dhima, Florian Mandija

**Affiliations:** Department of Physics, University of Tirana, Tirane, Albania; Departament of Physics, University of Tirana, Shkoder, Albania

**Keywords:** Temperature effect, Electrical resistivity, Cellular polypropylene, Activation energy

## Abstract

In this paper, we determine the surface and volume electrical resistivity of the 50 μm thick cellular polypropylen (VHD50), for the temperature range 393–453 K. For this we use a contemporary methodology, which consist of a voltage measurement across the sample, with a known current flowing through it. This methodology includes a three-electrode system, which allows us to estimate the resistivity of the samples, based on their corresponding total resistances. The electric fields applied for a time interval of 1 min are of the order of 200 *kVm*^−1^. The order of magnitude of surface and volume electrical resistivity is 10^13^ Ω and 10^11^ Ωm, respectively. For both types of the resistivity, the temperature dependence is an increasing or decreasing exponential function, depending on the type of the activation energy, (its average value for the temperature range mentioned above is 41,20 *kJmol*^−1^), totally confirmed by the corresponding theoretical interpretation, conditioned by the ionic conduction.

The methodology and equipment used, as well as the satisfying accordance with the results, found out directly or indirectly with the consulted literature, confirm the high accuracy of experimental measurements.

## Introduction

Electrical properties of polymers are extremely important, because they represent a subject naturally involved in varied applications (Sessler 1987). Electrical resistivity is a very important parameter among the electrical properties of polymers, which characterizes a range of properties of insulating materials, such as their homogeneity, location of impurities, difussion rate of charges through them, etc. (Vila & Sessler 2001; 1993). A special type of polymer, with numerous applications nowadays, is the celular polypropylen (VHD 50) (Herrmann 2005; Boech 2007). It is used in various environments; thus we have considered it important to study its electrical properties as a function of temperature.

We note that the level of porosity also affects on electrical resistivity of polypropylene (Chand & Sharma 2011).

### Determination of electrical resistivity in the dependence of temperature

#### Model used

The model used is the celular polypropylen (VHD 50), 50 *μm* in thickness. As a result of the increasing demand by capacitor manufacturers, as well as to product films with better mechanical and electrical properties, as well as thermodynamic strength, the first trials with polypropylene (VHD 50) were started by the end of 1998.

The celular polypropylen (VHD 50) is biaxially oriented, with a regular structure of isotactic polymer chains – unlike atactic or syndiotactic polymer chains, it has appropriate characteristics to produce thin films of polypropylene type, (Herrmann 2005). Its special external characteristic is the white color produced by the compact cellular structure and is also formulated to be suitable for use with cold seal coatings.

Among its most specific characteristics we can mention: high resistance to tear and puncture; excellent resistance to low temperatures; dimensionally stable under conditions of varying humidity; high gloss face that contributes to good print presentation giving good impression of hygiene and cleanliness; very preferable for cold seal coating applications; protection against light induced fat oxidation due to the light barrier in the UV range; while the films produced by it have low water vapor permeability, resistance to oil and grease, and also are physiologically harmless.

The cellular polypropylene (VHD 50) is highly preferred for food packaging and meets the specific requirements on health and safety, being in total accordance with FDA and EC regulations, which includes the specific “German Food and Commodity Act” and the current recommendations of the BgVV, (Boech 2007).

#### Measurement method

Determination of electrical surface (*ρ*_*s*_) and volume (*ρ*_*v*_) resistivity of the model has been performed by measuring, respectively, its electrical surface (*R*_*s*_) and volume(*R*_*v*_) resistances, when it is placed in a setup made of three metallic electrodes. The electrode system that we used to perform the measurements, is made up according to the German standard, (Vila & Sessler 2001; 1993), as well as with the recommendations of (Jonassen et al. 1979; Nansson & Jonassen 1978). The geometry of the electrode system allows us to calculate the coefficients that relate electrical resistances with the above mentioned electrical resistivities. We used Model 600 B Electrometer (Instruction manual 2013), (Keithley Instruments), for measuring the surface resistivity. In the experiments with constant current, the electrometer measures the voltage in the unknown model, when we know the current that flows through it. The voltage is proportional to the model resistance. As the resistances to be measured were mostly above 10^11^ Ω, the FAST method (Nansson & Jonassen 1978), which is most preferred, was selected.

We used HERAEUS EW-BAL Thermostat Oven, 3a (Heraeus GmbH 2013), to fix or vary the temperature. When the desired temperature is reached, it is kept fixed by the current generated in the thermocouple. It also plays the role of a supplementary Faraday cage.

#### Determination of dependence of surface electrical resistance on temperature

**Figure 1:**
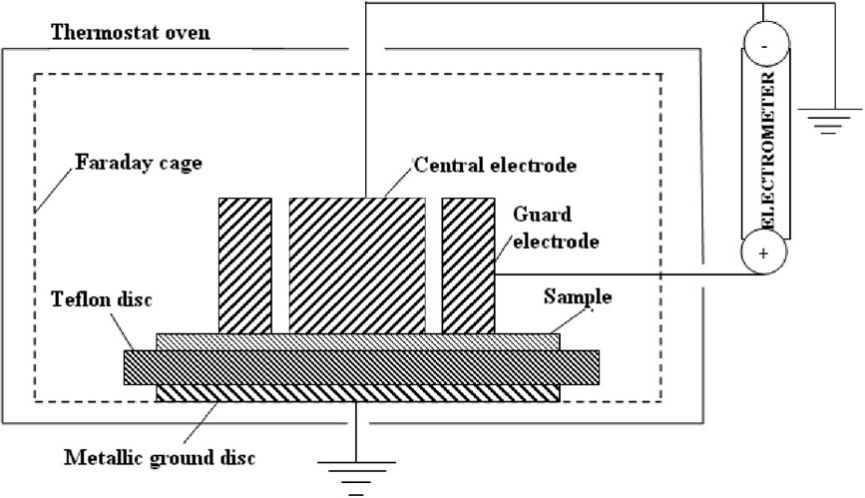
**Schematic diagram for measuring the surface electrical resistivity.**

The schematic diagram of experimental measurements for the determination of the surface electrical resistivity is presented in Figure 1. It is important for the guard electrode to have a positive potential in order to gather electrons on it.

The surface electrical resistivity, according to the proposed scheme, can be determined by the relation(Vila & Sessler 2001):
1

where *D*_2_and *D*_1_ are, respectively, the inner diameter of the guard electrode and the central electrode diameter, while *R*_*s*_ is the surface resistance experimentally determined by the electrometer.

The maximal error of the methodology and apparatuses used is 6%.

#### Determination of dependence of volume electrical resistance to temperature

**Figure 2:**
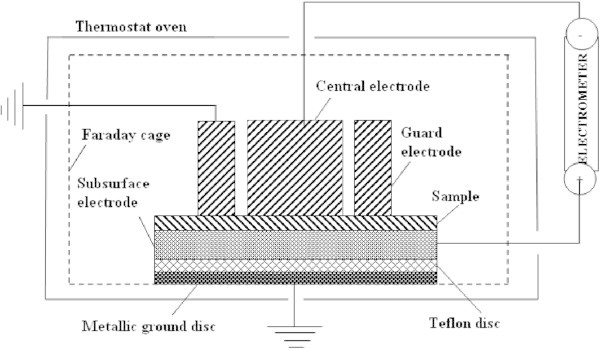
**Schematic diagram for measuring the volume electrical resistivity.**

The schematic diagram of experimental measurements for the determination of the volume electrical resistivity is presented in Figure 2. In this case it is important for the electrode under the surface of the model to have a positive potential in order to gather electrons on it.

Calculation of the volume electrical resistivity (*ρ*_*v*_) from the measured volume resistance (*R*_*v*_), (), involves, among other things, the real surface *S* of measurement electrode, but, since the model material is homogeneous and isotropic, fringes of the current lines in the area of the edges, may effectively increase the dimensions of the electrode diameter (1993; Vila et al. 2005) by the amount *Bg***,** where,


while *d* and *g* are, respectively, the model thickness and the distance between central and guard electrode. Therefore, the exact “effective” area (*S*_*eff*_) of contact between the central electrode and the model is:
2

where, *D*_1_ is the central electrode diameter. Thus, the volume electrical resistivity is determined by the relation:
3

But, in our experimental conditions  << 1; thus we can write:
4

The volume resistivity *R*_*v*_ is measured with the same apparatus, as well as the surface resistance.

The maximal error of the methodology and apparatus used is 6.4%.

## Experimental results, surface electrical resistivity

### Surface electrical resistivity

**Figure 3:**
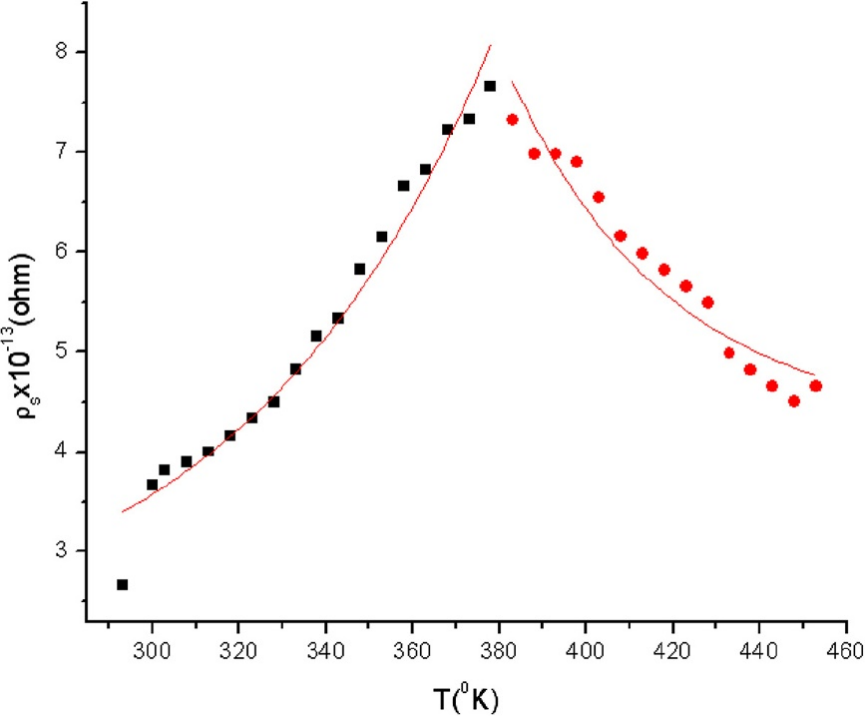
**Exponential dependence of the surface electrical resistivity on temperature.**

The experimental values of dependence: *ρ*_*s*_ = *f*(*T*), as well as the approximate curves of exponential type:
5

are presented in Figure 3.

The exponential approximation process can be divided into two intervals. The first involves the temperature range 293 *K* − 378 *K*, where *a*_1_ = 188.67*Ω* and *b*_1_ = − 1212 *K*, while the second involves the temperature range 383*K* − 453 *K*, where *a*_2_ = 0, 27*Ω* and *b*_2_ = 1285 ± 61 *K*.

### Volume electrical resistivity

**Figure 4:**
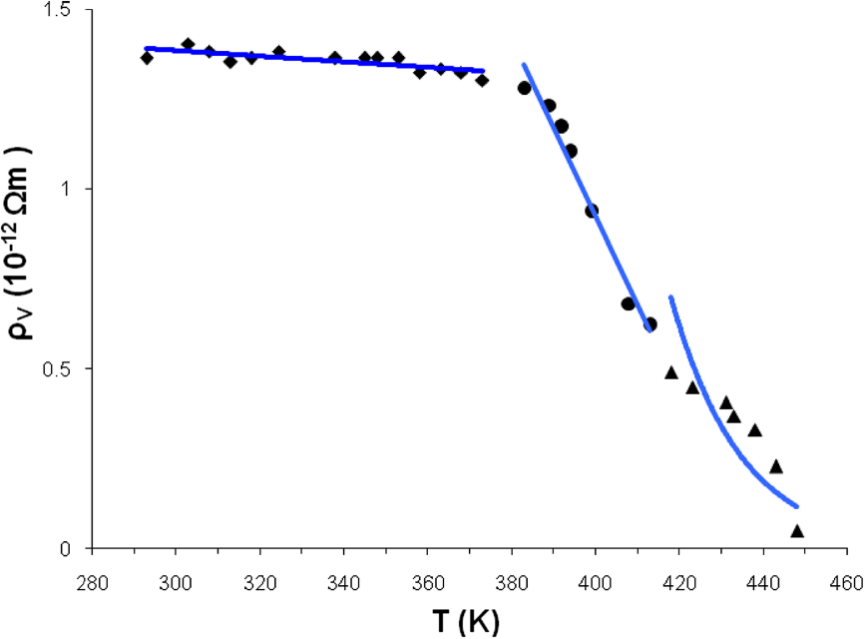
**Exponential dependence of the volume electrical resistivity on temperature.**

The experimental values of dependence:


as well as the approximate curves of exponential type:
6

are presented in Figure 4.

The exponential approximation process can be divided into three intervals. The first involves the temperature range 293 *K* − 373 *K*, where *α*_1_ = 11, 24*Ωm* and *β*_1_ = 63 ± 16 *K*. The second one involves the temperature range 383 *K* − 413 *K*, where *α*_2_ = 2, 43×10^− 7^*Ωm* and *β*_2_ = 6901 ± 1473 *K*. The third, involves the temperature range 418 *K* − 448 *K*, where *α*_3_ = 7, 29×10^− 18^*Ωm* and *β*_3_ = 17738 ± 3768 *K*.

## Discussion

### Interpretation of experimental results

Charge transport in materials is conditioned by electrons, ions or colloidal charged particles. Electrical conductivity increases with the increasing of the concentration*n*_*i*_, charge *q*_*i*_ and mobility *χ*_*i*_ of these charged particles:
7

The summation in the equation above is performed for all types of charge carriers.

Of the three types of electrical conductivity observed in polymers, for the interpretation of experimental results we will consider the electrical conductivity which is conditioned by the ions movement. The reason for this is that in our case the fields applied are weak (E≈ 2*kVcm*^−1^) and they extend in short time intervals (1min) (Sessler et al. 1986; Sawwa et al. 1980). Also the temperature range where the experiments are made is generally more than 300K, so the ionic conduction effect is more probable, because a combination of Schottky and Pool-Frenkel effects are observed in lower temperatures (Nevin & Summe 1981).

**Figure 5:**
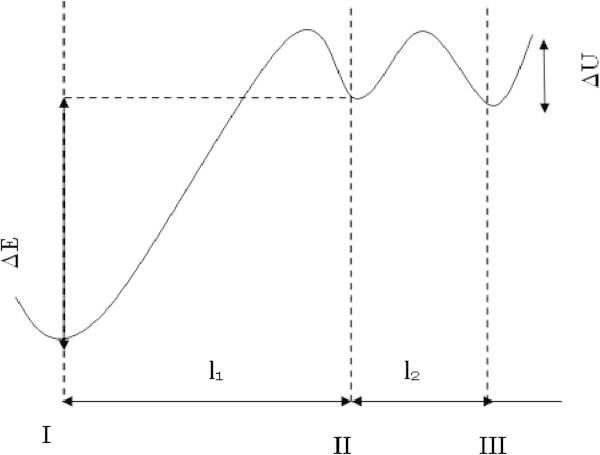
**The scheme of the potential barriers of ion movement in dielectric.**

In the: “bonded” state (not dissociated), the ion is in the *I* state with the lowest energy (Figure 5). As a result of thermal movement fluctuation, there is a possibility of “liberation” of a portion of the “bonded” ions and overcoming in their *II* state. Characteristic of the *II* state is the existence of vacancies in which the ion may be located. If the number of ions in the*I* state is *n*_0_, then, the number of ions in the *II* state, in conditions of an electric field applied, is (Sazhin 1970):
8

where, ∆*E* where is the difference in ion’s energy between *I* and *II* states, while *l*_1_ is the distance between *I* and *II* state. For the ion mobility in moving from *II* state to *III* state and beyond, in the field direction the following expression can be used (Nevin & Summe 1981):
9

where ∆*U*−the potential barrier between *II* and *III* equilibrium states; *l*_2_ − the distance between these states; *f*_0_− the frequency of vibrations in *II*and *III* states (Figure 5);

Knowing that the velocity of ions movement is *v* = *χE*, and the current density, *j* = *nqv* = *nqχE* = *γE*, derives *γ* = *nqχ*.

If the electrical conductivity is conditioned by the movement of a type of ion, then by neglecting the second term of (8), we obtain:
10

or
11

The first exponential term in equation (11) takes into account the “liberation” and ion movement under the influence of thermal movement. The second exponential term takes into account the influence of the field in the charge carriers concentration, while the multiplier , takes into account the influence of the field in the mobility of ions.

If, *l*_1_ = *l*_2_ = *l* (see Figure 5) we have:
12

and
13

By neglecting the term , the relation (11) can be written:
14

If we note  and *W*^∗^ = *ΔE* + *ΔU*, where W^*^ is the activation energy for a molecule, we obtain:
15

while arguing for a mole, the above relation can be written:
16

where *W*is the activation energy for a mole. The inverse of the above formula ultimately determines the electrical resistivity dependence of the temperature:
17

which is in full accordance with the fitting formula of experimental results.

### Exponential type functions and activation energy

Exponential approximations of experimental results are of two types, increasing and decreasing functions. The question is: How do we explain this?

Formula (17) shows clearly that the “key” to answering this question is the activation energy. It is usually identified as the energy barrier that must be surmounted to enable the occurrence of the bond redistribution steps required to convert reactants into products.

Its magnitude is identified as the difference between the energy of the molecules undergoing reaction and the overall average energy (Brown & Galweyl 1995). The activation energy may be positive, negative, or zero, depending on the complexity of the reaction being investigated. When the activation energy is negative, a standard interpretation of the observation is available: the reaction under investigation is multistep and involves at least one intermediate step (Turro et al. 1982). So, the type of reaction that clearly gives the change of activation energy sign is the multistep reaction. For a reaction with a “pre-equilibrium”, there are three activation energies to be taken into account; two of which refer to reversible steps of pre-equilibrium and one to the final step. The relative magnitudes of activation energies determine whether the total activation energy is positive or negative (Atkins 2006). We distinguish two types of reactions:

- Exothermic, associated with heat production and the activation energy, in this case, is negative (*W* < 0); b) endothermic, associated with heat absorption and activation energy, in this case, is positive (*W* > 0) .From the relation (16) we observe that with the increase of the temperature, for *W* < 0, the exponential  increases, while for *W* > 0, the exponential  decreases. This way the increasing or decreasing trend of exponential functions that give the temperature dependence of electrical resistivity, can be explained.From the graphs we see that, for the same model, there are different values of activation energy.

### Variation of activation energy and electrical resistivity

In the graph of the dependence of the surface electrical resistivity on temperature (Figure 3), one can clearly distinguish two zones:

In the first zone, we notice an exponential increase. In this case *W* = − 10, 09*kJmol*^−1^ < 0 , thus the reaction is exothermal. The temperature increase leads to an intensification of thermal movement of ions. In exothermal reactions, ions have enough energy to surmount the potential barrier and undergo reaction. This means that the number of ions which contribute to conductivity is reduced, thus the electrical resistivity will be increased.

The second zone corresponds to an exponential decrease.

In this case *W* = 10, 68*kJmol*^−1^ > 0, thus the reaction is endothermic. In endothermic reactions, ions do not have enough energy to surmount the potential barrier and easily undergo the reaction. This means that a large number of ions contribute to conductivity thus the electrical resistivity will be decreased.

In the graph of the dependence of the volume electrical resistivity on temperature (Figure 4), we can distinguish three exponentially decreasing zones:

This means that reactions that occur are characterized by a positive activation energy *W* > 0 and with the increase of the temperature, the exponential  decreases. In the first zone (where *W*_1_ = 0, 52*kJmol*^−1^ > 0) ions do not have enough energy to undergo the reaction**.** Thus, the electrical resistivity decreases.

In the second zone *W*_2_ = 57, 40*kJmol*^−1^ > *W*_1_ the energy barrier that ions have to surmount to undergo the reaction, is higher.

So, the number of ions that contribute to conductivity is larger than in the first zone and therefore the electrical resistivity continues to decrease, but the decrease happens more rapidly than in the first zone.

In the third zone, *W*_3_ = 147, 54*kJmol*^−1^ > *W*_2_ > *W*_1_, the energy barrier is higher than in two other zones. This means that the number of ions that are able to undergo the reaction is very small. The conductivity increases. The electrical resistivity continues to decrease, but the decrease happens more rapidly than in the first and second zones.

### Estimation of activation energy

Although the fundamental object of our study is not the activation energy, we see important to stop on an estimation of it, comparing relations (5) or (6) of experimental approximations with relation (17) of theoretical interpretation:
18

The estimations show that in case of cellular polypropylene (VHD 50), the average activation energy, for the temperature range between 293 and 453 K, is *W* = 41, 2*kJmol*^−1^. This is in full accordance with the order of the values of activation energies, obtained by other researchers, for polypropylene: *W* = 38.94*kJmol*^−1^ (Eckstein et al. 1998), *W* = 40, 61 − 41, 87*kJmol*^−1^ (Pearson et al. 1988) and *W* = 41, 87*kJmol*^−1^ (Fujiyama et al. 2002). This fact clearly confirms the accuracy of experimental measurements.

## Conclusions

The dependence of surface and volume electrical resistivity on temperature of cellular polypropylene (VHD 50) is determined by modern methodology and apparatus. Experimental results are approximated with exponential type functions, a fact that is in full accordance with the theoretical interpretations that are based on the ionic conduction. In the case of surface electrical resistivity, the exponential dependence is at first increasing and then decreasing, a fact that is explained by the activation energy, which in this case can be positive or negative. While in the case of volume electrical resistivity, the exponential dependence is just decreasing, due to the activation energy, which is only positive. And it is precisely this energy that gives us information on the tendency of ions to undergo reactions, or to contribute in the electrical resistivity of the special type of studied polypropylene, with many practical applications.
